# P-1783. Differences in stewardship strategies across hospitals performing well and poorly on a risk-adjusted metric for post-discharge antibiotic use

**DOI:** 10.1093/ofid/ofae631.1946

**Published:** 2025-01-29

**Authors:** Daniel J Livorsi, James Merchant, Hyunkeun Cho, Matthew B Goetz, Bruce Alexander, Brice Beck, Michihiko Goto

**Affiliations:** University of Iowa Carver College of Medicine, Iowa City, Iowa; Iowa City VA Health Care System, Iowa City, Iowa; University of Iowa Carver College of Medicine, Iowa City, Iowa; VA Greater Los Angeles Healthcare System, Los Angeles, California; Iowa City VA Medical Center, Iowa City, Iowa; Iowa City VA Health Care System, Iowa City, Iowa; University of Iowa/Iowa City VAMC, Iowa City, Iowa

## Abstract

**Background:**

Antibiotic overuse at hospital discharge is common, and it is unclear which antibiotic stewardship (AS) strategies are effective at improving post-discharge antibiotic-prescribing. In this study, we compared how hospitals’ AS processes differed based on their performance on a risk-adjusted metric for post-discharge antibiotic use.

**Figure 1.**
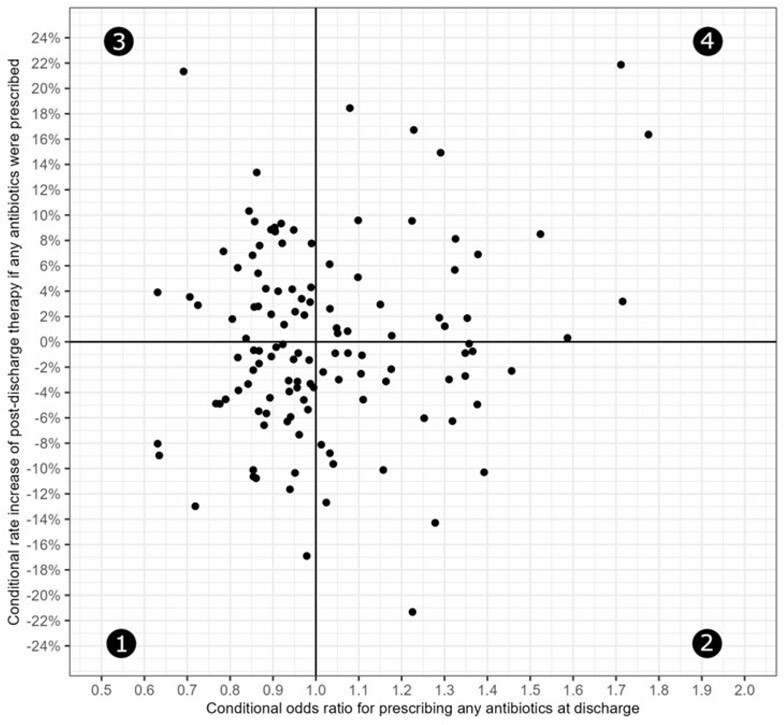
Risk-adjusted comparison of post-discharge antibiotic-prescribing frequency and duration across 124 VA hospitals Group 1 hospitals (n=40) had less frequent post-discharge antibiotic-prescribing and used shorter post-discharge antibiotic duration. Group 2 hospitals (n=26) had more frequent post-discharge antibiotic-prescribing and used shorter post-discharge antibiotic durations. Group 3 hospitals (n=34) had less frequent post-discharge antibiotic-prescribing and used longer post-discharge antibiotic durations. Group 4 hospitals (n=24) had more frequent post-discharge antibiotic-prescribing and used longer post-discharge antibiotic durations.

**Methods:**

We performed a retrospective study of discharges to home from 124 acute-care VA hospitals. Discharges occurred 6 months before/after a mandatory hospital-level AS survey (11/10/20); patients who received ≥ 30 days of post-discharge antibiotics were excluded. We built a zero-inflated negative binomial mixed-model with two random intercepts for each hospital to predict post-discharge oral antibiotic exposure and duration. Key model covariates included patient comorbidities, discharge diagnoses of infection, and inpatient antibiotic duration. Using the predicted random intercepts, hospitals were categorized into 4 groups. Next, we used a multinomial logistic regression model to compare how often AS processes, as reported in the survey, were used at hospitals across these groups.

**Table 1.**
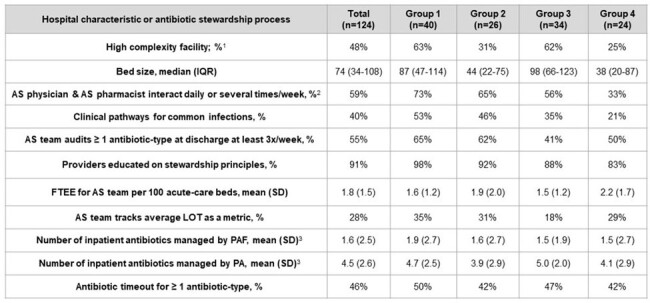
Characteristics of 124 VA hospitals, stratified by a hospital’s performance on a risk-adjusted metric for post-discharge antibiotic use Abbreviations: AS = antibiotic stewardship; FTEE = full time employee equivalents; IQR = interquartile range; LOT = length of therapy; PA = prior authorization; PAF = prospective audit-and-feedback; SD = standard deviation. (1) Facility complexity scores are created by the VA Healthcare Analysis and Information Group. Hospitals are scored according to their patient population, clinical services (e.g., intensive care unit and surgery services), education and research. A score of 1a and 1b is categorized as the highest complexity. Lower complexity facilitates are scored as 1c, 2, or 3. (2) This survey question was dichotomized to compare stewardship physicians and stewardship pharmacists who interact daily or several times a week versus teams that interact weekly, monthly or less frequently than monthly. (3) This variable ranges from 1-10 and captures whether certain antibiotics were managed by prior authorization (PA) or prospective audit-and-feedback (PAF). Each additional antibiotic managed with one of these strategies increased the PA or PAF score by 1, except as noted: vancomycin (intravenous), daptomycin, oral/IV linezolid (each formulation = 0.5 points), piperacillin-tazobactam, cefepime, ceftazidime, anti-pseudomonal carbapenems, ertapenem, ciprofloxacin + levofloxacin oral/IV (each 0.5), and moxifloxacin oral/IV (each 0.5).

**Results:**

There were 399,234 discharges; 17.2% received post-discharge antibiotics (median duration 6). Hospital performance on the metric is shown in Figure 1, and Table 1 shows hospital characteristics, based on the group. Forty (32.3%) hospitals were in group 1, *i.e.*, they prescribed antibiotics less frequently than expected at discharge and used shorter post-discharge duration. Twenty-four (19.4%) hospitals were in group 4, *i.e.*, they prescribed antibiotics more often than expected at discharge and used longer post-discharge duration. Compared to group 1 hospitals, hospitals in group 4 were less likely to report interactions between their AS physician and AS pharmacist(s) every day or several times per week (0.17, 95% CI 0.05-0.64). No other AS processes significantly differed between the groups (Table 2).

**Table 2.**
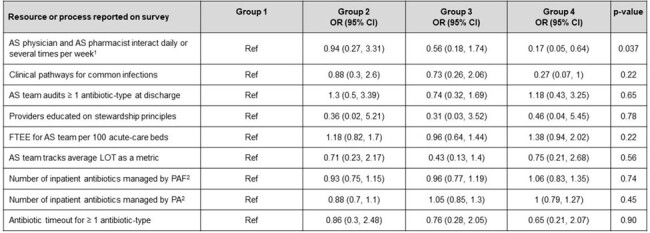
Odds ratios for a hospital within each group reporting the use of specific antibiotic stewardship processes, based on a multinomial logistic regression model Abbreviations: AS = antibiotic stewardship; FTEE = full time employee equivalents; LOT = length of therapy; OR = odds ratio; PA = prior authorization; PAF = prospective audit-and-feedback; Ref = reference group. (1) This survey question was dichotomized to compare stewardship physicians and stewardship pharmacists who interact daily or several times a week versus teams that interact weekly, monthly or less frequently than monthly. (2) The odds ratio reflects the effect on the odds of increasing the number of antibiotics managed with this strategy by a single unit (i.e., managing 10 antibiotics by PA versus managing only 9 by PA).

**Conclusion:**

The findings of this mixed-methods study suggest that routine AS physician-pharmacist collaboration may be important to reducing antibiotic overuse at discharge. Future studies should more precisely measure AS processes to better understand how these are associated with differences in hospital performance at this transition of care.

**Disclosures:**

**Daniel J. Livorsi, MD**, Merck: Grant/Research Support **Michihiko Goto, MD MSCI**, Merck: Grant/Research Support

